# Application of genome-wide insertion/deletion markers on genetic structure analysis and identity signature of *Malus* accessions

**DOI:** 10.1186/s12870-020-02744-2

**Published:** 2020-11-30

**Authors:** Xuan Wang, Fei Shen, Yuan Gao, Kun Wang, Ruiting Chen, Jun Luo, Lili Yang, Xi Zhang, Changpeng Qiu, Wei Li, Ting Wu, Xuefeng Xu, Yi Wang, Peihua Cong, Zhenhai Han, Xinzhong Zhang

**Affiliations:** 1 College of Horticulture, China Agricultural University, Beijing, China; 2 Beijing Agro-biotechnology Research Center, Beijing Academy of Agriculture and Forestry Sciences, Beijing, China; 3 Research Institute of Pomology, Chinese Academy of Agricultural Science, Xingcheng, Liaoning China; 4 Present Address: Shaanxi Haisheng Fruit Industry Development Co., Ltd., Shaanxi Xian, China; 5 College of Information and Electrical Engineering, China Agricultural University, Beijing, China

**Keywords:** *Malus*, InDel, Bud sports, Genetic structure, Germplasm

## Abstract

**Background:**

Apple (*Malus* ssp.), one of the most important temperate fruit crops, has a long cultivation history and is economically important. To identify the genetic relationships among the apple germplasm accessions, whole-genome structural variants identified between *M. domestica* cultivars ‘Jonathan’ and ‘Golden Delicious’ were used.

**Results:**

A total of 25,924 insertions and deletions (InDels) were obtained, from which 102 InDel markers were developed. Using the InDel markers, we found that 942 (75.3%) of the 1251 *Malus* accessions from 35 species exhibited a unique identity signature due to their distinct genotype combinations. The 102 InDel markers could distinguish 16.7–71.4% of the 331 bud sports derived from ‘Fuji’, ‘Red Delicious’, ‘Gala’, ‘Golden Delicious’, and other cultivars. Five distinct genetic patterns were found in 1002 diploid accessions based on 78 bi-allele InDel markers. Genetic structure analysis indicated that *M. domestica* showed higher genetic diversity than the other species. *Malus* underwent a relatively high level of wild-to-crop or crop-to-wild gene flow. *M. sieversii* was closely related to both *M. domestica* and cultivated Chinese cultivars.

**Conclusions:**

The identity signatures of *Malus* accessions can be used to determine distinctness, uniformity, and stability. The results of this study may also provide better insight into the genetic relationships among *Malus* species.

**Supplementary Information:**

The online version contains supplementary material available at 10.1186/s12870-020-02744-2.

## Background

Apple (*Malus* ssp.), one of the most commonly cultivated fruit crops, supports many local economies in temperate zones. *Malus* is extremely rich in diversity, with 25 to 78 species in the genus depending on the taxonomic classifications [51, 56]. High levels of interspecific hybridization occur naturally, which generates genetic admixtures, contributing to the diversity within the genus [6, 7, 12]. In addition to the natural diversification of the genus, anthropogenic activities, including selection and cross breeding, have led to approximately 10,000 cultivars worldwide [8, 21, 65]. Identification of the distinctness of the germplasm would be beneficial to the successful conservation and efficient utilization of genetic resources.

The genetic variability and allelic diversity in these accessions are usually examined to reveal their distinctness. Identification of population structure and kinship within germplasm collections is a fundamental prerequisite for identifying robust marker-trait associations [68]. There are also possibly duplicates, synonyms, homonyms, or materials with missing names that must be carefully examined among the living collections [40]. For example, a previous study identified 330 apple cultivars or abandoned trees that could be either grafted clones or ‘own rooted seedlings’ using nine SSR markers [24]. In addition, the test for distinctness, uniformity, and stability (DUST) is a statutory requirement to release a new cultivar (International Union for the Protection of New Varieties of Plants (UPOV) Convention Articles 5–9, 991)[64]. Limited by the fact that traditional field tests are time-consuming, laborious, and greatly influenced by the environment, DNA markers are used in DUST in many species [25, 58]. Therefore, it is also necessary to develop an efficient marker-assisted DUST protocol in *Malus* plants.

Owing to the co-dominant inheritance and because they are often multi-allelic, simple sequence repeat (SSR) markers have been widely used in apples to evaluate genetic diversity, population structure, and to analyze parentage [16, 23, 30, 32, 40, 43, 65]. However, the disadvantages of SSRs are frequently reported. The instability of SSRs increased dramatically with plant age [22]. Certain chemicals or radiation may cause DNA double-strand breaks, and the repair of these breaks usually results in small insertions or deletions (InDels) at the break site. These InDels presumably contribute to the instability of SSRs [22]. Errors can also be found in the documented parentage of some accessions by comparing the SSR profiles to show parent-offspring similarity [16, 46].

Single nucleotide polymorphism (SNP) markers are commonly used in large-scale, high-throughput automated detection of genetic variation because of their large number and wide distribution in the genome [44, 67]. A previous study used an 8 K apple SNP array [5] to identify cryptic relationships between accessions, analyze population structure, and calculate the linkage disequilibrium in apple [68]. Similarly, 3704 confident SNPs were used to analyze a core collection of cider and dessert French apple cultivars [34].

In addition to SSR and SNP markers, InDels have been recognized as an ideal source for marker development due to their high-density, co-dominance, robust stability, and genotyping efficiency [28, 74]. InDel markers have been used to identify the specificity of germplasm resources and provide information for breeding in chickpea (*Cicer arietinum* L.) [28], cotton (*Gossypium hirsutum* L.) [74], pepper (*Capsicum spp.* L.) [26], *Carapa guianensis* [63], mung bean (*Vigna radiata* (L.) Wilczek) [35], and cucumber (*Cucumis sativus* L.) [36]. In addition, InDel markers have been successfully used to distinguish somatic variations in apple [33], although InDel markers have not been as widely used in apple as SSRs and SNPs.

The objectives of the current study were to develop a set of stable co-dominant InDel markers and to identify *Malus* accessions. Genome-wide InDels were robustly used for analysis of distinctness, genetic structure, genetic composition, and the parentage of 1251 *Malus* accessions. The results provided insight into *Malus* germplasm resources and may facilitate the future utilization of germplasm in apple breeding.

## Results

### Genome-wide structural variant (SV) calling and selection of InDel markers

**Fig. 1:**
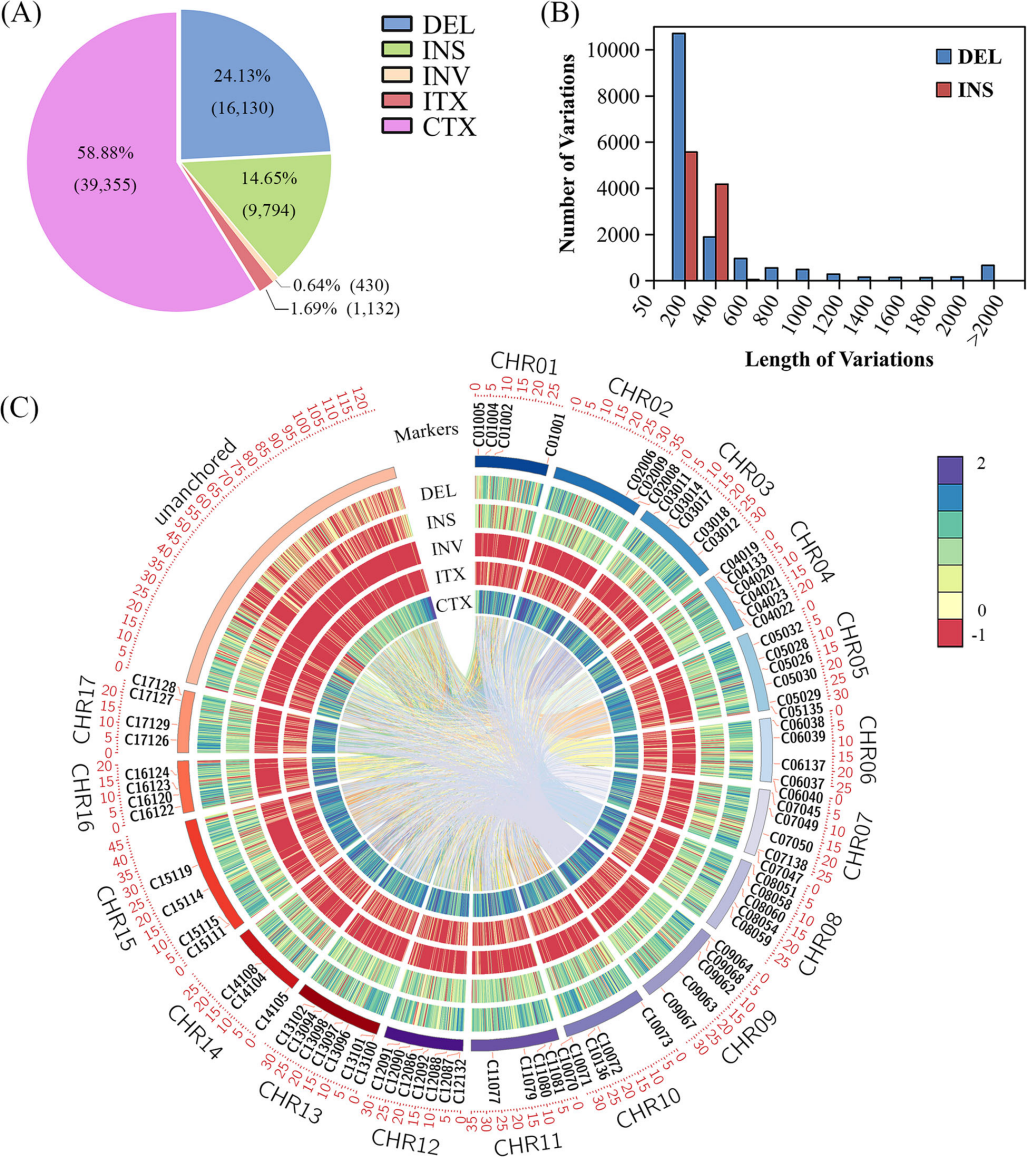
The properties of structural variants (SVs) and the genome-wide distribution of insertion/deletion (InDel) markers selected between apple (*Malus domestica*) cultivars ‘Golden Delicious’ and ‘Jonathan’. **a** Proportion of each type of SV. DEL: deletion; INS: insertion; INV: inversion; ITX: intra-chromosomal translocation; CTX: inter-chromosomal translocation. Percentages and numerals in brackets indicate the proportion and number of different types of SV, respectively. **b** The fragment length of INS and DEL. **c** The genome-wide distribution of SVs and the 102 selected InDel markers. The rectangles in the outer-most whirl represent the chromosomes, the SVs cannot be reliably unanchored to any chromosome were marked by ‘unanchored’. The chromosome number and the physical position are labeled on the edges of the plot. The inner whirls represent the distribution of DEL, INS, INV, ITX, and CTX on each chromosome. The lines connecting in the center of the figure indicate the corresponding positions before and after the shifts due to ITX and CTX. The value corresponding to the chromaticity bar represents the logarithm of the number of SVs in the range of 0.2 Mb on the chromosome. ‘-1’ on the chromaticity bar corresponds to no SVs in the range of 0.2 Mb

The next generation resequencing data from the two apple founder cultivars, ‘Jonathan’ and ‘Golden Delicious’, resulted in an average read depth of 43.44 and have been deposited in the NCBI Sequence Read Archive (SRA) with the accession number PRJNA392908 [60]. A total of 66,841 genome-wide SVs between ‘Jonathan’ and ‘Golden Delicious’ were obtained using the apple genome v1.0 as reference [69], including 16,130 deletions (DEL), 9794 insertions (INS), 430 inversions (INV), 1132 intra-chromosomal translocations (ITX), and 39,355 inter-chromosomal translocations (CTX) (Fig. 1a-c). Our results showed that InDels were more well-distributed on chromosomes than the other types of SVs (Fig. 1c). The length of the majority of DEL (78.15%) and INS (99.53%) ranged from 50 bp to 400 bp (Fig. 1b), indicating that InDels are more representative for genome-wide marker development because of their large number and frequent distribution throughout the genome.

**Fig. 2:**
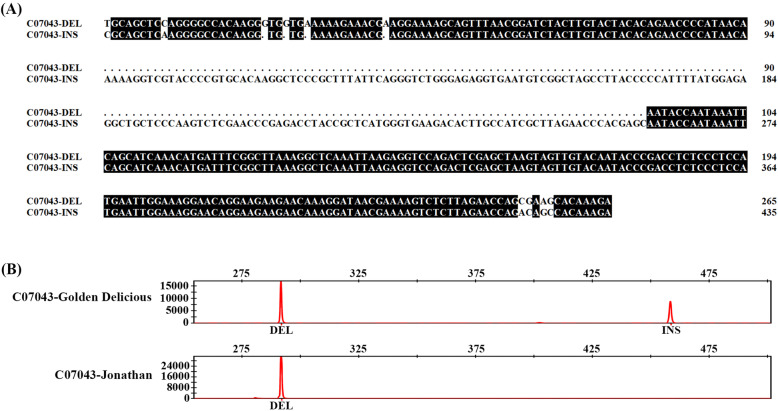
The sequences and genotypes of selected insertion (INS)/deletion (DEL)(InDel) markers (C07043 as an example) were validated by Sanger sequencing (**a**) and capillary electrophoresis (**b**) using the apple cultivars ‘Golden Delicious’ and ‘Jonathan’. In panel **b**, the numbers on the vertical axis show relative fluorescence intensity, whereas those on the horizontal axis indicate approximate fragment size in base pairs

Of the 170 InDels chosen throughout the genome (10 per chromosome), 102 were validated for further analyses (example of validated Indel in Fig. 2a and b, list of validated InDels in Table S1). These InDels were combined into nine fluorescence multiplex PCR groups; each group contained three to 24 InDel markers (Table S2).

### Genotyping of the selected InDel markers and generation of identity signatures of accessions

Of the 1251 *Malus* accessions included in this study, 942 exhibited unique genotype combinations (Table S3). Three hundred and nine accessions shared genotype combinations with at least one of the other accessions, which comprised of 76 distinct patterns (Tables S3 and S4). Sixty-one accessions were found to be synonyms (including 2 with known alternative names), 78 were homonyms, and 8 were replicated collections (Table S5). The genotypes of two tetraploids, ‘Zumi Crab 4x’ (B-21) and ‘Gala 4x’ (XC-4), were the same as their diploid progenitors, ‘Zumi Crab’ (B-30) and ‘Ruihong’ (QD-25), respectively (Table S4). There were 199 accessions that were mutants of nine known cultivars (Table S4) and 22 accessions had registration errors or incorrect names (Table S5). Excluding synonyms and accessions with errors or incorrect names, 1018 accessions were identified as unique by the 102 InDel markers (Table S3). The InDel identity signature was then generated for each of these 1018 accessions with a 2-dimensional bar code (QR code) conveying the 102 InDel marker genotypes (Supplementary File S1).

### Identification of somatic mutants

**Fig. 3:**
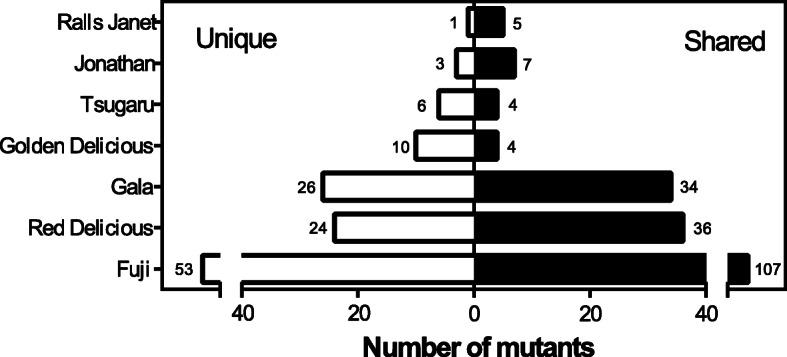
The proportions of bud sports from cultivated apple cultivars that were distinguishable using the 102 insertion/deletion markers

In this study, 331 of the included 981 *Malus domestica* Borkh. accessions were bud sports derived from commercially used cultivars. These mutants included 160 bud sports of ‘Fuji’, 60 bud sports of ‘Red Delicious’, 60 bud sports of ‘Gala’, and 40 bud sports derived from each of the following cultivars: ‘Golden Delicious’, ‘Tsugaru’, ‘Jonathan’, and ‘Ralls Janet’ (Fig. 3).

Fifty-three (33.1%) of the 160 ‘Fuji’ mutants were distinguished from the other ‘Fuji’ bud sports. The remaining 107 ‘Fuji’ bud sports were classified into 11 subgroups, each composed of two to 75 accessions (Fig. 3; Tables S3 and S4). Similarly, among the ‘Red Delicious’ bud sports, the genotypes of 24 (40.0%) of the 60 accessions were distinct from the other ‘Red Delicious’ bud sports, whereas the other 36 bud sports shared three genotype combinations (Fig. 3; Tables S3 and S4). Twenty-six (43.3%) of the 60 ‘Gala’ bud sports were distinct using these InDel markers; 34 bud sports showed six genotype combinations (Fig. 3; Tables S3 and S4). Regarding the bud sports of ‘Golden Delicious’, ‘Tsugaru’, ‘Jonathan’, and ‘Ralls Janet’, 10 of the 14 (71.4%), six of the 10 (60.0%), three of 10 (30.0%), and one of the six (16.7%) were uniquely distinguished, respectively (Fig. 3; Tables S3 and S4).

Furthermore, we compared the marker genotypes of 24 bud sports derived from ‘Fuji’, ‘Gala’, and ‘Red Delicious’ with the corresponding wildtype cultivars. The wildtype cultivar (e.g. ‘Starking’) of a certain bud sport (e.g. ‘Starkrimson’) refers to the cultivar from which the bud sport has been selected. A wildtype cultivar (e.g. ‘Starking’) can sometimes also be a bud sport of an older cultivar (e.g. ‘Red Delicious’). The genotypes of the 102 InDel markers of five bud sports were identical to the corresponding wildtype cultivars (Table S6). Polymorphisms in at least one marker were detected in 19 bud sports compared with the corresponding wildtype cultivars.

### Genetic composition of the InDel markers

**Fig. 4:**
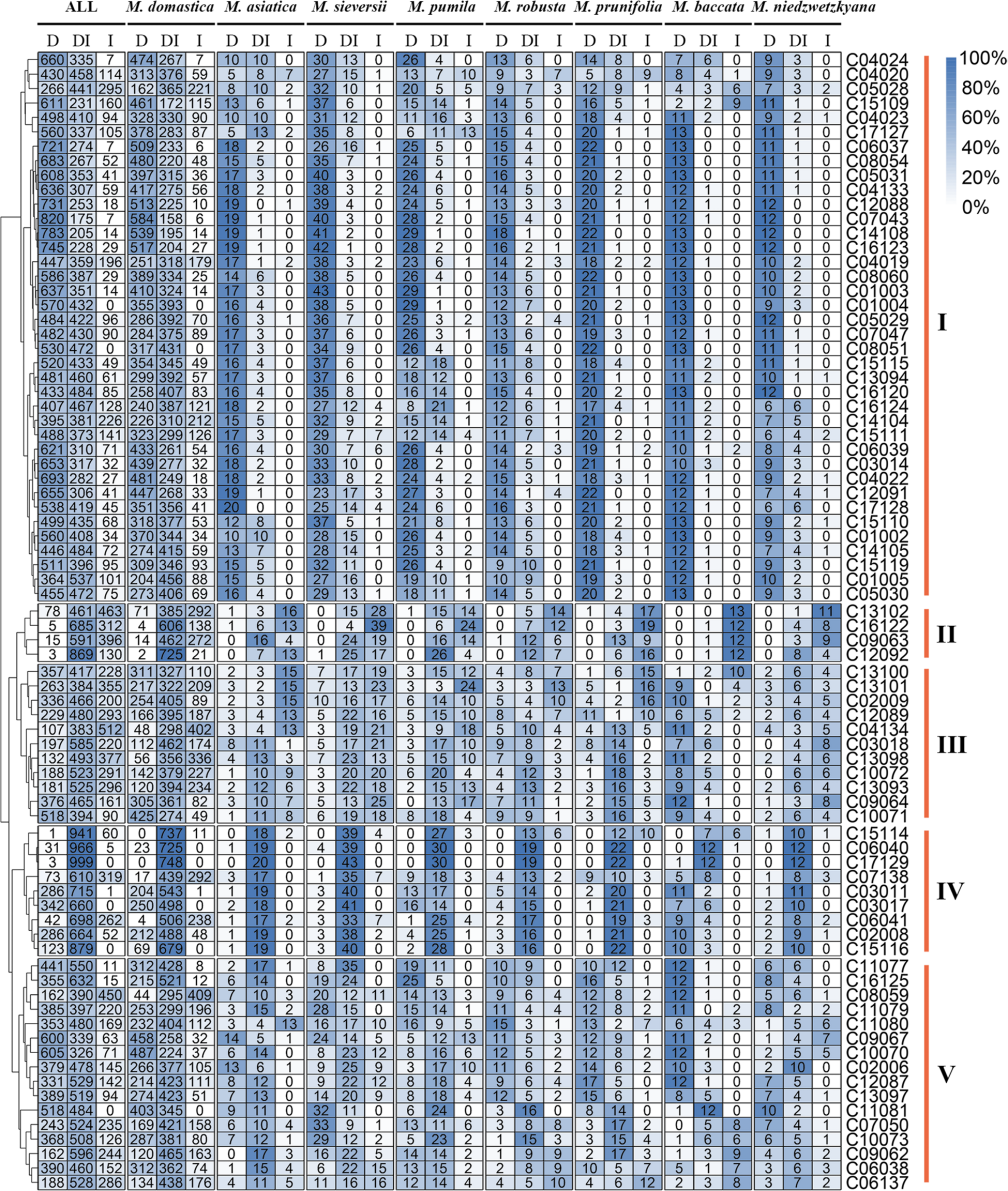
The genotype frequency of 78 insertion (I)/deletion (D) markers in 1002 *Malus* accessions. The numerals indicate the number of accessions with a certain genotype pattern. The marker names are given on the right margin, and the colors represent the genotype frequency

Five genotype distribution patterns were detected among the 78 biallelic InDel markers using the unique 1002 diploid accessions (Fig. 4; Table S7). Pattern I (38 markers) was characterized by the relatively low frequency (7.0%) of homozygous INS in *M. domestica* compared with the extremely high frequency (71.9%) of homozygous DEL genotypes. In other species than *M. domestica*, much lower frequency (2.0%) of homozygous INS genotypes were detected and the frequency of genotypes with heterozygous DEL:INS was also relatively low (Fig. 4; Table S7). Four markers showed pattern II genotype distribution, where the homozygous DEL genotypes were detected in low frequencies in *M. domestica* and were rare or completely absent in other species (Fig. 4; Table S7). Pattern III (11 markers) exhibited no obvious distortion in marker genotype frequency distribution but few marker/species combinations complied with Hardy-Weinberg equilibrium (Fig. 4; Table S7). Pattern IV (9 markers) was characterized by extremely high frequency (80.0%) of heterozygous DEL:INS genotype in every species, except for five markers in *M. baccata,* of which the frequency of homozygous DEL genotypes was higher (Fig. 4; Table S7). Pattern V (16 markers) showed the same pattern as pattern III (Fig. 4; Table S7).

### Parentage analysis

The parentage analysis allowed for the identification of the parent-offspring relationships among the accessions. The parentage of 66 cultivars was confirmed (Table S8), and the documented parentage of six accessions was found to be incorrect (Table 1). The cultivar ‘53–205’, which was believed to be a hybrid from ‘Jonathan’ × ‘Golden Delicious’, was found to be a first-generation offspring of ‘Jonathan’ × ‘Miyazaki Spur Fuji’. Two supposed full-siblings, ‘33–018’ and ‘33–101’, hybrids with the parents ‘Zisai Pearl’ × ‘Golden Delicious’, were identified to be half-siblings instead; the male parents were ‘Miyazaki Spur Fuji’ and unknown, respectively. Similarly, the parentage of ‘H5–101’, ‘50–32’, and ‘62–45’ was corrected (Table 1). The unknown parents of seven cultivars were hypothesized based on the parent-offspring relationships. For example, ‘Harlikar’ was selected in Japan from an open pollinated progeny of ‘Golden Delicious’. Herein, we propose that the paternal parent was ‘Oregon Spur II’ or a related somatic mutant of ‘Red Delicious’ (Table 1).

**Table 1:** Newly proposed parentage of 13 *Malus domestica* accessions (> 0.98 confidence)

Accession number	Accession name	Documented parentage	Newly proposed parentage
YX-33-018	33–018	Zisai Pearl × Golden Delicious	Zisai Pearl × **Miyazaki spur Fuji**
YX-33-101	33–101	Zisai Pearl × Golden Delicious	Zisai Pearl × **Unknown**
YX-53-205	53–205	Jonathan × Golden Delicious	Jonathan × **Miyazaki Spur Fuji**
CL-5	H5–101	Golden Delicious × Jonathan	Golden Delicious × **Fuji**
CL-4	50–32	Miyazaki Spur Fuji × Starkrimson	Miyazaki Spur Fuji × **Unknown**
XY-68	62–45	Hanfu × Yueshuai	**Fuji** × Yueshuai
WH-8	Harlikar	Golden Delicious × Unknown	Golden Delicious × *Oregon spur II*
BK-DANXIA	Danxia	Golden Delicious × Unknown	Golden Delicious × *Red Delicious*
BK-YG	Yoko	Golden Delicious × Unknown	Golden Delicious × *Jonathan*
7--23	Youyi	Jonathan × Unknown	Jonathan × *Summer Pearmain*
B-2	Jiping 1	Mato 1 × Unknown	Mato 1 × ***Gala***
4--7	Fuhong	Unknown	*Jonathan* × *Red Astrachan*
8--19	Shennong 2	Unknown	*Golden Delicious* × Unknown

Note: The italic font indicates the newly proposed parentage which was unknown before, and the bold font indicates the error in the documented parentage was corrected

### Genetic structure analysis

A genetic structure analysis was generated based on 173 accessions of seven *Malus* species (Table S9). All seven *Malus* species showed relatively low inbreeding coefficients, indicating a low level of population structure within these species (Table 2). Both the highest expected (*He*) and the highest observed heterozygosity (*Ho*) were obtained in *M. domestica*. Conversely, the lowest level of genetic diversity was detected in *M. baccata*, as shown by the lowest *He* and *Ho* (Table 2). Similarly, the highest and the lowest average number of effective alleles were observed in *M. domestica* and *M. baccata*, respectively (Table 2).

**Table 2:** Summary of genetic variation in seven *Malus* species

	Na	Ne	I	Ho	He	F
***M. domestica***	2.000	1.774	0.618	0.508	0.429	−0.173
***M. sieversii***	1.987	1.544	0.484	0.367	0.320	−0.097
***M. asiatica***	1.987	1.543	0.481	0.404	0.318	−0.172
***M. pumila***	2.000	1.581	0.502	0.396	0.335	−0.122
***M. robusta***	2.000	1.598	0.532	0.383	0.354	−0.063
***M. prunifilia***	1.936	1.467	0.418	0.315	0.274	−0.042
***M. baccata***	1.795	1.277	0.294	0.180	0.181	−0.010

Na: average number of alleles; Ne: average number of effective alleles; I: Shannon’s diversity index; Ho: observed heterozygosity; He: expected heterozygosity; F: inbreeding coefficient

To estimate the genetic differentiation between *Malus* species, pairwise differentiation (*Fst*) values were calculated and all *Fst* values were highly significant (*P* < 0.001) (Table 3). The highest level of genetic differentiation was found between *M. baccata* and all of the other species (*Fst* = 0.061–0.129). The differentiation between *M. domestica* and the six other species (*Fst* = 0.033–0.129) was higher compared to the other five species (*Fst* = 0.02–0.037) (Table 3).

**Table 3:** Pairwise differentiation (*Fst*) between the seven *Malus* species

	M. ***domestica***	***M. sieversii***	***M. asiatica***	***M. pumila***	***M. robusta***	***M. prunifilia***	***M. baccata***
***M. domestica***	–						
***M. sieversii***	0.051	–					
***M. asiatica***	0.054	0.028	–				
***M. pumila***	0.046	0.028	0.034	–			
***M. robusta***	0.033	0.033	0.028	0.033	–		
***M. prunifilia***	0.074	0.033	0.020**	0.037	0.026	–	
***M. baccata***	0.129	0.107	0.091	0.104	0.069	0.061	–

Note: All *Fst* values were significant at *P* < 0.0001, except for the number marked with ** which indicates *P* < 0.005

**Fig. 5:**
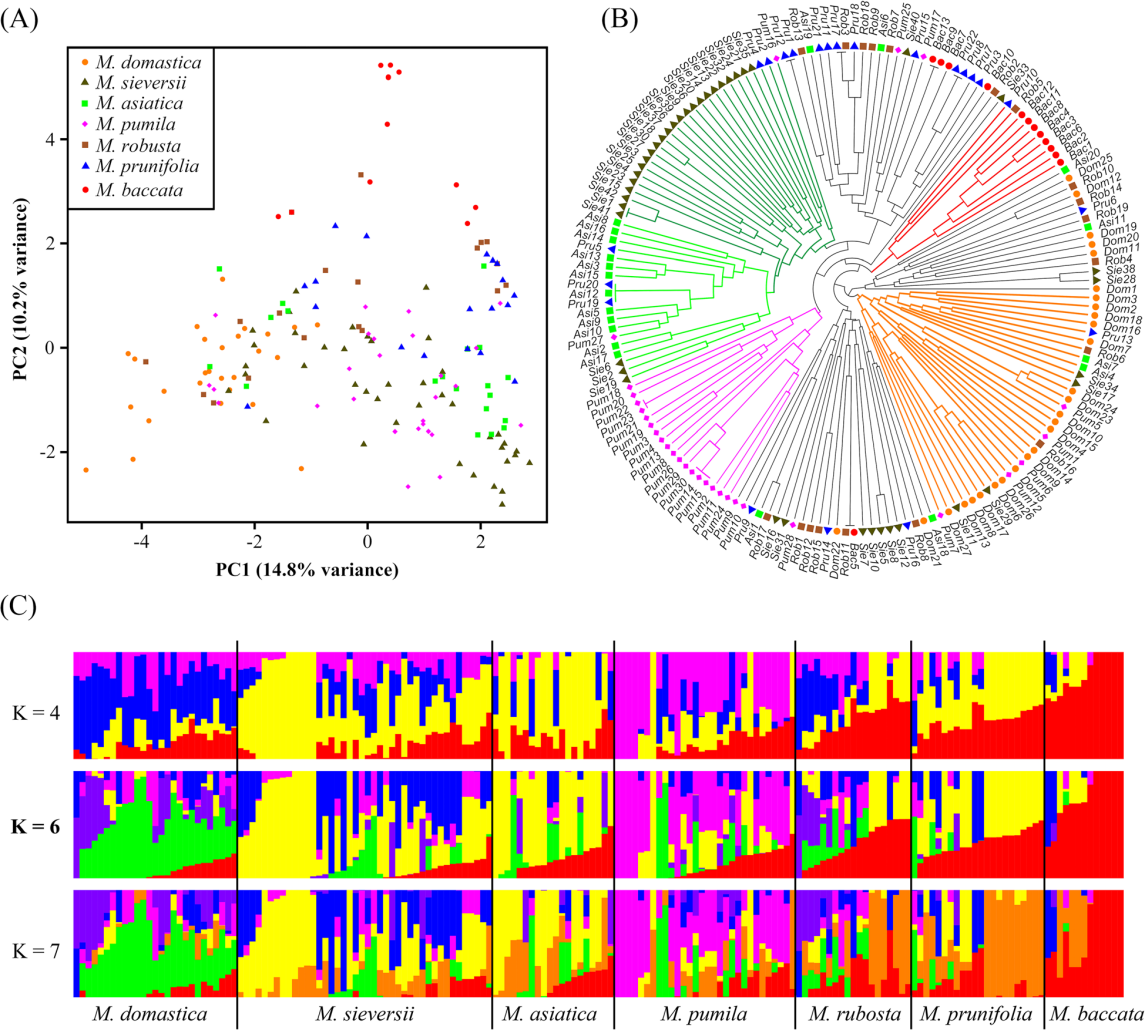
Genetic structure analyses depicting the relationships among seven *Malus* species. **a** Principal component analysis of 173 apple accessions from the seven species. **b** A phylogenetic analysis using insertion/deletion markers. Refer to panel A for the legend. **c** STRUCTURE analysis of 173 *Malus* accessions

Genetic discrimination between the seven species was confirmed through a multivariate Principal Component Analysis (PCA) (Fig. 5a). In the bi-dimensional plot, we found that the two species *M. domestica* and *M. baccata* were completely separate (Fig. 5a). *M. asiatica* was divided into two groups; one was distributed in the lower right corner adjacent to *M. sieversii*, while the other was admixed with *M. domestica*. Most accessions of *M. pumila* admixed with *M. sieversii,* whereas *M. robusta* and *M. prunifolia* were scattered with other species (Fig. 5a).

Relationships among the accessions of the seven different *Malus* species were also depicted using a phylogenetic analysis (Fig. 5b). Our results showed that most accessions of *M. sieversii, M. pumila,* and *M. baccata* formed separate clades. Twelve of the 20 *M. asiatica* accessions were typically found to be closely related to *M. sieversii*. *M. robusta* and *M. prunifolia* were largely clustered in the same clade (Fig. 5b). *M. baccata* was found to be basal to the other six species, whereas *M. domestica* was at the distal end of the phylogenetic tree (Fig. 5b). A subset of 13 *M. sieversii* and 12 *M. robusta* accessions were clustered close to *M. domestica*. Several accessions of *M. asiatica*, *M. robusta*, *M. prunifolia*, *M. sieversii*, and *M. pumila* were scattered in the large *M. domestica* clade (Fig. 5b).

Finally, relationships among the *Malus* species were explored using ADMIXTURE cross-validation, which indicated that K = 6 was a sensible modeling choice; the other inflection points were K = 4 and K = 7 (Figure S1). Thus, the three Q estimates (K = 4, 6, and 7) were plotted separately (Fig. 5c). At both K = 6 and K = 4, *M. domestica*, *M. sieversii*, *M. pumila*, and *M. baccata* were clustered into separate gene pools. *M. sieversii* differentiated into two subdivisions, one of which (blue) exhibited introgression into *M. domestica* and *M. robusta*. The other subdivision (yellow) of *M. sieversii* showed apparent gene flow into *M. asiatica* and *M. prunifolia* (Fig. 5c). Introgression was also detected from *M. baccata* into other species, especially *M. prunifolia* and *M. robusta*. When K = 7, *M. prunifolia* clustered into a separate gene pool and showed gene flow (orange) into *M. robusta* (Fig. 5c).

## Discussion

Benefited from the high quality assemblies of the apple genome [13, 69, 75], large scale of SNP and InDel markers can be easily obtained [48]. In this study, we detected a total of 25,924 InDels between the two cultivars, ‘Jonathan’ and ‘Golden Delicious’. These InDels provided a large reservoir for high performance PCR-based DNA markers for the *Malus* genus [41, 66]. One hundred and two of these InDel markers were applied in this study to the following analyses in *Malus* accessions.

### The application of genome-wide InDel markers on the genetic structure analysis of *Malus* accessions

Seventy-eight bi-allele InDel markers were used in investigating the relationship between the seven *Malus* species. Lower *He* and *Ho* values, as well as lower average number of effective alleles were detected in the other six species than in *M. domestica* with the lowest values detected in *M. baccata*. Although these lower levels of *He*, *Ho*, and *Ne* could indicate lower levels of genetic diversity in these species, low values could also be observed because the InDel markers were developed from two *M. domestica* cultivars. The lowest *He, Ho* and *Ne* values were detected in *M. baccata* indicating a low level of genetic diversity in this species. Additionally, the phylogenetic analysis and the structure analysis showed less genetic relatedness of *M. baccata* accessions to the other species. The two subdivisions of *M. sieversii*, however, showed gene introgression into *M. domestica* or *M. robusta* and *M. asiatica* or *M. prunifolia*, respectively. These data were highly consistent with the bi-directional gene flow of *M. sieversii*, which is believed to be the common ancestral species of *M. domestica* and ancient Chinese apple cultivars [15, 69]. *M. domestica* was domesticated in Central Asia from *M. sieversii*, but as it migrated westwards, it hybridized with the European crabapple *M. sylvestris* and/or *M. orientalis*, from which modern apples are descended [10]. However, the DNA ITS1 sequences and genomic regions used in previous studies were not informative for discriminating the samples of *M. sylvestris*, *M. sieversii*, and *M. domestica* [8, 59]. When ancient *M. domestica* moved eastwards, it hybridized with several local wild or semi-cultivated relatives to created Chinese domesticated landrace cultivars, such as ‘Nai’, which is highly similar to *M. sieversii* and contained a small signature from other wild apple species [15, 39]. The close genetic relatedness of *M. asiatica* or *M. prunifolia* to *M. baccata* and *M. sieversii* identified in this study supports the previous hypothesis that Chinese native species, such as *M. asiatica* and *M. prunifolia*, are very likely to be hybrids between *M. baccata* and *M. sieversii* [15].

Genetic diversity in domesticated species is often affected by intentional artificial selection and unintentional genetic bottlenecks [9]. Over the last 800 years, *M. domestica* showed no significant reduction in genetic diversity [23], which can possibly be explained by the wild-to-crop introgression [12]. Interspecific hybridization may be an important mechanism for germplasm diversification, and similar genes across multiple species underlies parallel/convergent phenotypic evolution between taxa [53]. The highest level of genetic diversity among the seven *Malus* species was observed in *M. domestica*, indicated by the highest *He* and *Ho*. During domestication and evolution, both the modern deliberate selection and past natural selection may gradually change the genetic composition of a species [53]. We found the genetic composition differed among the InDel markers and *Malus* species.

In this study, the low inbreeding coefficients of all the seven species were consistent with the high level of gametophytic self-incompatibility in *Malus* [14, 42, 73]. The lowest inbreeding coefficient was detected for *M. domestica* and *M. asiatica*, which could be explained by the artificial selection for cultivars with high levels of heterozygosity [12].

The highest inbreeding coefficient observed among the seven species in this study was in *M. baccata*. This observation is likely an artifact from our marker development panel, which consisted only of *M. domestica* accessions. Because *M. baccata* is rather distantly related to *M. domestica* [12], our markers were likely not as informative in this species.

In this study, we found relatively high differentiation between *M. domestica* and the other species. While wild-to-crop gene flow may occur naturally, anthropogenic factors, such as apple production and the variations in apple flower visitors, significantly impact wild-to-crop gene flow [9]. We observed that several accessions of *M. asiatica*, *M. robusta*, *M. prunifolia*, *M. sieversii*, and *M. pumila* scattered in the *M. domestica* clade (Fig. 5b). This is similar to previous findings that showed high levels of introgression from *M. domestica* detected in *M. orientalis* (3.2% of hybrids), *M. sieversii* (14.8%), and *M. sylvestris* (36.7%) [11]. Conversely, gene flow from domesticated-to-wild accessions or escapes from cultivated *M. domestica* threatens the fitness and the genetic integrity of wild relatives; therefore, it is important to conserve wild germplasm resources [6, 20].

### The application of genome-wide InDel markers to delineate the identity signature of *Malus* accessions

Identity signatures of 1018 *Malus* accessions were created as QR codes using the 102 InDel markers in this study. These QR codes can not only used for DUST within *Malus*, but also can distinguish some of the bud sports of apple cultivars. Early studies attempted to distinguish bud sports of apple cultivars with amplified fragment length polymorphism markers; however, the efficiency was low [37, 76]. Recent studies have had limited success distinguishing clonal mutants because the high levels of clonality or homogeneity among cultivars derived from bud sports [12]. A previous study used two InDel markers to efficiently and specifically distinguish ‘Fuji’ and its somatic variant ‘Benishogun’ from four other bud sport cultivars [33]. In this study, the 102 InDel markers discriminated successfully 33.1, 40.0, 43.2, and 71.4% of bud sports of ‘Fuji’, ‘Red Delicious’, ‘Gala’, and ‘Golden Delicious’. There would be three reasons why the bud sports cannot be fully distinguishable. The first is that some bud sports are genetically identical due to the parallel or reproducible occurrence of somatic variations in fruit crops [3, 29]. The second aspect that hinders the genetic identification of bud sports is chimeric forms of somatic variation in fruit crops [17–19, 70]. Epigenetic variations may be the third source of clonal differences that have been difficult to be detected genetically [45, 61, 71].

### The application of genome-wide InDel markers for lineage tracing of *Malus* accessions

Many apple cultivars, such as ‘Red Delicious’, ‘Golden Delicious’, and ‘Ralls Janet’, originated from chance seedlings and one or even both parents of these cultivars are unknown [44]. Lineage tracing back of cultivars with unknown parentage has been pioneered in ‘Honeycrisp’ by SSR markers and SNP linkage maps [4, 27]. Most recently, the parent-offspring relationships of 1400 apple cultivars were analyzed with whole-genome SNPs [44]. By using the 102 InDel markers in this study, the previously reported parentage of 66 cultivars was corrected, whereas previously unknown parents of seven cultivars, such as ‘Harlikar’, were identified (Table 1). To elucidate the pedigree or the genetic background of cultivars with unknown parentage, the cost of using these 102 InDel markers should be lower than the available SNP arrays ([2, 5, 27, 38];). However, it would be impossible for these InDels to compose haplotypes, as has been done in some previous studies (e.g. [27]), duo to the marker density being too low.

## Conclusions

One hundred and two stable co-dominant long InDel markers were developed in *Malus*. Identity signatures of 1018 *Malus* accessions were created as QR codes using these markers. The QR codes can not only be used for DUST, but also can efficiently distinguish some bud sports of apple cultivars. These markers were also used in the analysis of parent-offspring relationship to determine the previously unknown parentage. The application of these InDel markers on the genetic structure analysis also provided insight into the genetic relationships among *Malus* species.

## Methods

### Plant materials

We sampled and analyzed a collection of 1251 *Malus* accessions, including 981 accessions of *M. domestica* Borkh., 49 accessions of *M. sieversii* (Ledeb.) Roem., 20 accessions of *M. asiatica* Nakai, 31 accessions of *M. pumila* Mill., 21 accessions of *M. robusta* Rehder, 25 accessions of *M. prunifolia* (Wild.) Borkh., 13 accessions of *M. baccata* (L.) Borkh., and 111 other species (Table S3). All the plant materials are originally collected and possessed by China Agricultural University and Chinese Academy of Agricultural Science, respectively. The experiments on plants including field investigation and sample collection were performed under institutional guidelines in accordance with local legislation. Young leaf samples were collected and stored on silica gel. The genomic DNA was extracted using the modified CTAB protocol [62].

### Calling of SV from previous resequencing data of ‘Jonathan’ and ‘Golden delicious’

SV were called using Delly (version 0.8.1) software [54]. The BAM files from the cultivars ‘Jonathan’ (SRX4380657) and ‘Golden Delicious’ (SRX4380658) ([60]; https://www.ncbi.nlm.nih.gov/sra/?term=PRJNA392908) were fed into the Delly call function with default parameters to call SVs. The distribution of the obtained SVs in the genome of the accessions was presented using Circos (version 0.69–8) software [31].

### Selection and genotyping of InDel markers for all accessions

One hundred and seventy InDels with 50–400 bp polymorphic fragments were selected to develop InDel markers, ten InDels were selected in each of the 17 chromosomes. The InDel fragments were validated between ‘Jonathan’ and ‘Golden Delicious’ by Sanger sequencing and capillary electrophoresis analysis. Only the markers that were confirmed to produce unique, valid amplified products and were used for further analysis.

The multi-PCR forward primers of each InDel markers were labeled with the fluorescent dyes FAM, HEX, NED, and PET (Table S2). Multi-PCR was performed in a final volume of 10 μL containing 1 μL of DNA template (200 ng), 1 μL of primer mix, 4 μL of 2.5 × Master Mix I (Beijing Microread Genetic Co., Ltd., Beijing, China), and 4 μL of double distilled water (ddH_2_O). The thermocycler conditions were set as follows: pre-incubation at 95 °C for 5 min; followed by 35 cycles of 30 s for denaturing at 95 °C, 90 s for annealing at 55 °C, and 90 s for elongation at 72 °C; and a final extension 72 °C for 15 min. Amplified products were stored at 12 °C until analysis with an ABI3730 XL sequencing system (Applied Biosystems, Foster City, CA, USA). Fragment and sizing analyses were carried out using GeneMapper v.5.0 software (Applied Biosystems, Foster City, CA, USA), and chromatograms were independently read by two operators.

The identity signature of the accessions was represented by the unique genotype combination of the 102 InDel markers. Then the genotype information from the accessions was used to create a QR code using an online tool (https://cli.im/).

### Identification of genetic composition of the InDel markers

For the genetic composition analysis, only the unique 1002 diploid accessions were used and the 78 biallelic InDel markers were selected from all markers and were used in the analysis. The results of genetic composition were visualized by a heatmap using the pheatmap package (https://www.rdocumentation.org/packages/pheatmap/versions/1.0.12) with default clustering method in R.

### Genetic structure analysis

For the genetic structure analysis, the 78 bi-allele InDel markers were used and 173 *Malus* germplasm accessions with unique genotype combinations were selected, including 27 randomly chosen from *M. domestica*, and all accessions in relative species (42 *M. sieversii*, 20 *M. asiatica*, 30 *M. pumila*, 19 *M. robusta*, 22 *M. prunifolia*, and 13 *M. baccata*) (Table S9). Known polyploid accessions were not included here to ensure bi-allele genetic composition. *He* and *Ho* were estimated with GenAlEx 6.5 [49, 50]. *Fst* between species was assessed in exact tests using GENEPOP 4.0 [55, 57].

To elucidate the genetic relationship among accessions, a PCA was performed using the pca3d (version 0.10) package in R [72]. A phylogenetic tree was built using the ape (version 5.3) package in R [47]. A population structure analysis was performed using the block relaxation algorithm implemented in ADMIXTURE (version 1.3) software [1]. We generated the associated support files using PLINK (version 1.90) software [52].

### Parentage analysis

To determine the parentage of some *M. domestica* cultivars, the parent-offspring relationships of accessions with one or two unknown parents were analyzed based on the genotype data of the 102 InDel markers using a custom Python script, AppleParentage1.0 software (https://github.com/wangx321/AppleParentage1.0). The confidence parameters were set to > 0.98 (Threshold = 1).

## Supplementary Information

**Additional file 1: Supplementary File S1.** The QR code giving the molecular ID of 1018 *Malus* accessions.

**Additional file 2: Supplementary Table S1.** Sequences, PCR product sizes and primers of the 102 InDel markers.

**Additional file 3: Supplementary Table S2.** Fluorescent labelled multiplex PCR matching schemes of the 102 insertion/deletion markers.

**Additional file 4: Supplementary Table S3.** Genotypes of the 102 InDel markers for 1,251 *Malus* accessions.

**Additional file 5: Supplementary Table S4.** *Malus* accessions with shared genotype combinations of the 102 InDel markers.

**Additional file 6: Supplementary Table S5.** Synonyms, homonyms and other *Malus* accessions with incorrect names detected by the 102 InDel markers.

**Additional file 7: Supplementary Table S6.** Comparison of genotypes of InDel markers between bud sports and corresponding wild-type *Malus domestica* cultivars.

**Additional file 8: Supplementary Table S7.** Genotype and allele frequencies of 78 bi-allele InDel markers in 1,002 diploid accessions from eight *Malus* species.

**Additional file 9: Supplementary Table S8.** Parentage analysis of 66 *Malus domestica* cultivars using InDel markers.

**Additional file 10: Supplementary Table S9.** *Malus* accessions used for genetic structure analysis.

**Additional file 11: Supplementary Figure S1.** Cross-validation plot for the InDel dataset.

## Data Availability

All DNA re-sequencing reads are freely available and have been upload to Sequence Read Archive (SRA) database (https://www.ncbi.nlm.nih.gov/sra/?term=PRJNA392908) already.

## Abbreviations

CTAB: Cetyltrimethylammonium bromide
CTX: Inter-chromosomal translocation
DEL: Deletion
DUST: Test for distinctness, uniformity, and stability
*Fst*: Fixation index ‘F-statistics’
*He*: Expected heterozygosity
*Ho*: Observed heterozygosity
InDel: Insertion and deletion
INS: Insertion
INV: Inversion
ITS1: Internal transcribed spacer 1
ITX: Intra-chromosomal translocation
PCA: Principal component analysis
PCR: Polymerase chain reaction
QR code: 2-dimensional bar code
QTL: Quantitative trait loci
SNP: Single nucleotide polymorphism
SRA: Sequence Read Archive
SSR: Simple sequence repeat
SV: Structural variant
