# Supplementary material for: Application of genome-wide insertion/deletion markers on genetic structure analysis and identity signature of Malus accessions
Source: BMC Plant Biol. 2020 Nov 30;20:540. doi: 10.1186/s12870-020-02744-2 (PMC7708918; doi:10.1186/s12870-020-02744-2)
Supplement: Supplementary file 1 — Additional file 1: Supplementary File S1. The QR code giving the molecular ID of 1018 Malus accessions. [file 12870_2020_2744_MOESM1_ESM.pdf]

Supplementary File S1

The QR code giving the molecular ID of 1018 *Malus* accessions.

01-001<br>(YX-01-001)	01-121<br>(YX-01-121)	02-009<br>(YX-02-009)	03-010<br>(YX-03-010)	03-06-04<br>(SY-8)	03-111<br>(YX-03-111)	04-033<br>(YX-04-033)	04-087<br>(YX-04-087)
06-056<br>(YX-06-056)	07-115<br>(BK-1)	08-034<br>(YX-08-034)	09-037<br>(YX-09-037)	09-079<br>(YX-09-079)	10-010<br>(YX-10-010)	10-182<br>(YX-10-182)	107/06<br>(GZ-2)
11-037<br>(YX-11-037)	11-206<br>(YX-11-206)	117/06<br>(GZ-3)	119/06<br>(GZ-4)	11906<br>(B-41)	12-206<br>(YX-12-206)	13-025<br>(YX-13-025)	13-26W<br>(CL-1)
147<br>(B-38)	15-26<br>(XY-8)	16-155<br>(YX-16-155)	16-157<br>(YX-16-157)	17-023<br>(YX-17-023)	17-199<br>(YX-17-199)	19-147<br>(XYZ-9)	21-005<br>(YX-21-005)
21-018<br>(YX-21-018)	22-186<br>(YX-22-186)	23-127<br>(CL-2)	23-42<br>(XY-62)	23-63<br>(XY-61)	23#<br>(B-37)	26105<br>(B-16)	26-34<br>(XY-5)
27-003<br>(YX-27-003)	28-253<br>(BK-28-253)	29-176<br>(YX-29-176)	30-001<br>(YX-30-001)	33-018<br>(YX-33-018)	33-101<br>(YX-33-101)	33-151<br>(YX-33-151)	4-23<br>(BK-4-23)
4354<br>(BK-4)	Gala 4x<br>(TA-1)	Hanfu 4x<br>(TA-2)	500g<br>(21--9)	50-30<br>(CL-3)	50-32<br>(CL-4)	51-007<br>(YX-51-007)	51-031<br>(YX-51-031)
51-077<br>(YX-51-077)	51-102<br>(YX-51-102)	51-165<br>(YX-51-165)	51-166<br>(YX-51-166)	51-209<br>(YX-51-209)	52-049<br>(YX-52-049)	52-151<br>(YX-52-151)	52-160<br>(YX-52-160)
53-040<br>(YX-53-040)	53-205<br>(YX-53-205)	54-001<br>(YX-54-001)	54-188<br>(YX-54-188)	55-006<br>(YX-55-006)	55-023<br>(YX-55-023)	55-042<br>(YX-55-042)	56-081<br>(YX-56-081)
58-036<br>(YX-58-036)	58-089<br>(YX-58-089)	58-144<br>(YX-58-144)	58-177<br>(YX-58-177)	58-211<br>(YX-58-211)	58-34<br>(XY-78)	59-086<br>(YX-59-086)	59-130<br>(YX-59-130)

600g Andong<br>(20--12)	62-45<br>(XY-68)	7-171<br>(XY-63)	7-211<br>(XY-4)	74-178<br>(XY-3)	77-34<br>(BK-77-34)	78-M18<br>(SY-1)	83-2<br>(MDJ-1)
95/06<br>(GZ-1)	99-1-29<br>(Z-22)	99-2-39<br>(Z-29)	99-2-58<br>(Z-17)	Aichi<br>(YT-19)	Aleksanader<br>(YT-83)	Anna<br>(Z-39)	Arkcharm<br>(YT-78)
Auraria<br>(YT-2)	Azwell<br>(BK-Azwell)	B009<br>(SX-22)	B68<br>(B-7)	Behene<br>(16--11)	Belgolden<br>(YT-43)	Bosh<br>(YT-47)	BP<br>(TA-3)
BP-176<br>(TA-4)	Britegold<br>(YN-19)	C37<br>(XYZ-11)	Canzy<br>(HS-17)	CG24<br>(BK-CG24)	CG3<br>(BK-CG3)	chadel<br>(YT-102)	charden<br>(YT-103)
Cornoet<br>(YT-17)	E3N2<br>(OR-3)	E4N1<br>(OR-4)	E4N2<br>(OR-5)	Elegia<br>(YT-4)	Elite<br>(BK-Elite)	Envy<br>(HS-15)	Envy<br>(BJ-12)
Florina<br>(BK-Florina)	Free Red Star<br>(YT-60)	Fuji KiKu<br>(BJ-2)	G30<br>(BK-G30)	G41<br>(TA-5)	G935<br>(TA-6)	Gala<br>(BK-gala)	Geaooza<br>(YT-23)
Generos<br>(22--7)	Gloster69<br>(BK-Gloster69)	GM256<br>(BK-GM256)	GM256<br>(XY-71)	GM310<br>(BK-GM310)	Gold milecnirum<br>(YT-15)	Golden Bell<br>(YT-77)	Gornan<br>(YT-75)
GS48<br>(24--3)	GS58<br>(23--21)	H5-101<br>(CL-5)	HAC-9<br>(BK-HAC-9)	HLWQ<br>(BK-HLWQ)	Holly<br>(BK-Holly)	HY<br>(B-33)	Jazz<br>(BJ-5)
Jonagored<br>(BK-Jonagored)	Jonathan-csan<br>(YT-35)	Jonathan-M41<br>(YN-17)	K10<br>(28--14)	K12<br>(28--8)	KOSZTELQ<br>(BK-KOSZTELQ)	M7<br>(BK-M7)	M9 pajam2<br>(Z-6)
Martinike<br>(YT-81)	Melfree<br>(YT-13)	Melrose<br>(YT-52)	Modi<br>(HS-7)	Mollies Del open<br>(YT-55)	Mrxl(robusta×Li<br>berty)<br>(YT-54)	NAKB clone<br>(Z-5)	NAKT M9 clone<br>(Z-13)
P16	P22	P60	Pionier	Prima	Prima×Sekaiichii	Priw	Reandra

(BK-P16)	(BK-P22)	(TA-7)	(BK-Pionier)	(BK-Prima)	(YT-105)	(YT-18)	(YT-50)
Redchif<br>(YT-79)	Regilndel<br>(YT-76)	Remo<br>(YT-58)	Renora<br>(YT-56)	Revbihola<br>(YT-51)	Rewena<br>(YT-53)	Rockit<br>(Z-53)	Rosegrow<br>(HS-16)
Rosmadzin<br>(YT-57)	Rubinola<br>(YT-44)	Scarlet<br>(BK-scarlet)	Sdw1<br>(BK-Sdw1)	Seokwang<br>(Z-23)	SH40-2 seedling<br>(QD-4)	SH6<br>(BK-SH6)	Siana<br>(YN-15)
siyana<br>(YT-1)	Sweetie<br>(Z-3)	Sweetle<br>(YT-82)	Szampion<br>(BK-Szampion)	T337<br>(BK-T337)	Telamon<br>(QD-20)	Teser<br>(YT-27)	Toppax apple<br>(YT-11)
Trajian<br>(BK-Trajian)	Tuskan<br>(YT-104)	W6N1<br>(OR-6)	W6S5<br>(OR-7)	W8S3<br>(OR-8)	WH-5<br>(WH-1)	wifos<br>(BK-wifos)	Y-1<br>(SX-21)
Y-2<br>(SX-25)	Y-3<br>(SX-26)	Znoga<br>(Z-51)	Alberta<br>(2--3)	Arkansas Black<br>(2--6)	Allington Pippin<br>(11--14)	Aliusitan<br>(YT-22)	Ace<br>(28--4)
Atlas<br>(17--13)	Ayiwaniya<br>(5--10)	Aifeng<br>(21--21)	Aihonghua<br>(B2-8)	Aizaohui<br>(1--2)	Idared<br>(YT-61)	Antalue<br>(21--1)	Oberkika<br>(13--17)
Aohong<br>(21--14)	Ozark Gold<br>(BK-AJ)	Aozhou 1<br>(TS-5)	Granny Smith<br>(24--4)	Baleng Crab<br>(B2-3)	Baleng seedling<br>14<br>(B-25)	Babskino<br>(13--12)	Buckeye Gala<br>(HS-5)
Beauty of Bath<br>(28--13)	Batougou 2<br>(BK-BTG2H)	Batougou<br>Aizhen<br>(BK-BTGAZ)	Batougou 2<br>(BK-BTG1H)	Batul<br>(10--7)	White Crab<br>(BK-BHT)	Bai Crab<br>(WH-2)	Bailuosi Malin<br>(12--4)
Lowtosh<br>(17--19)	Baifugao<br>(BK-BFG)	Bancroft<br>(17--23)	Sakatakei<br>Tsugaru<br>(10--5)	Banbishan Crab<br>(BK-BBSHT)	Banxiu Crab<br>(BJ-4)	Baoman<br>(10--2)	Baotou Linqin<br>(QD-31)
Hokudo<br>(BK-BD)	Beifang Xina<br>(28--16)	Kitanosach<br>(BK-BZX)	Bedan<br>(22--14)	Bell Poos<br>(21--7)	Benoni<br>(13--1)	Bisimake<br>(4--8)	Biaoguo Crab<br>(BK-PGHT)

Binlang<br>(SY-2)	Binzi<br>(BK-BZ)	Binzi (SW)<br>(BK-BZXN)	Bo 25<br>(7--17)	Bo 26<br>(12--18)	Bo 5<br>(10--23)	Adam<br>Mickewier<br>(9--19)	Buming<br>Kangbing<br>(BK-BMKB)
Bukowka<br>(12--11)	Bramley's<br>Seedling<br>(18--5)	Blengstid Gaurd<br>(20--2)	Cangjiang Crab<br>(BK-CJHT)	Caozigang<br>Yuanshuai<br>(BK-CZGYS)	Strawberry<br>(15--23)	Caoyuan Crab<br>(B2-1)	Caoyuan Crab<br>(QD-7)
Charden<br>(16--17)	Changhua<br>(XYZ-6)	Chaohongxing<br>(TS-3)	Chenyang<br>(Z-43)	Envy ?<br>(XYZ-12)	Baldwin<br>(19--10)	Rainier<br>(20--15)	Chu Tsugaru<br>(22--11)
Hatsuaki<br>(BK-CQ)	Duchess of<br>Oldenburg<br>(1--5)	Chuanling Crab<br>(BK-CLHT)	Chuizhi Fuji<br>(XY-27)	Chunxiang<br>(25--4)	Winesap<br>(20--16)	Cuihong<br>(9--13)	Darwin<br>(BK-DEW)
Da Baleng<br>(B-12)	Big Crab<br>(BK-DGHT)	Dagucheng<br>Baleng<br>(B1-5)	Daguo Jinhong<br>(BK-DGJH)	Twenty Ounce<br>(17--18)	Dalu 52<br>(3--12)	Olga<br>(SY-9)	Daxianguo<br>(BK-DXG)
Mother<br>(17--1)	Daye Crab<br>(BK-DYHT)	Daihao 261<br>(BK-DH261)	Dailv<br>(23--14)	Daihong<br>(QD-1)	Red June<br>(12--21)	Danxia<br>(BK-DANXIA)	De 14<br>(7--20)
De 2<br>(8--7)	De 4<br>(9--14)	De 6<br>(12--20)	De 8<br>(10--22)	Deqin Crab<br>(DQ)	line 13<br>(YN-24)	line 5<br>(YN-22)	line 6<br>(YN-23)
row 21<br>(YN-41)	row 9<br>(YN-29)	row 6<br>(YN-28)	row 10<br>(YN-30)	row 19<br>(YN-39)	row 16<br>(YN-36)	row 17<br>(YN-37)	row 13<br>(YN-33)
row 15<br>(YN-35)	row 11<br>(YN-31)	Oriental apple<br>(BK-DFPG)	Toko<br>(2--10)	Toko<br>(SY-5)	Dongqie Gala<br>(Z-44)	Dongxiangjiao<br>(11--5)	Donghongguo<br>(XC-DHG)
Donglimu<br>(SX-33)	Dounan<br>(B-40)	Huaguan Spur<br>(BK-DZHG)	Spur Mutsu<br>(22--1)	Dorsett Golden<br>(Z-16)	Duoyilu<br>(12--19)	Oregon Spur<br>II-red<br>(OR-1)	Oregon Spur<br>II-green<br>(OR-2)

Russian White apple<br>(B2-6)	Eluosi Daguo Shandingzi<br>(B3-3)	Russian apple<br>(B3-8)	E zhen 1<br>(QD-13)	E zhen 2<br>(QD-14)	E zhen 3<br>(QD-15)	E zhen 4<br>(QD-16)	E zhen 5<br>(QD-17)
Enqi<br>(27--13)	Enweier Golden<br>(5--6)	Fa 3<br>(16--8)	Fa 8<br>(BK-F8)	French apple<br>(9--11)	Skyline Spureme<br>(11--8)	Feixia<br>(31--1)	King of Tompkins County<br>(BK-FZY)
Fenhong Gala 44<br>(YT-67)	Pink Lady<br>(XN-FHNS)	Fengyan<br>(BK-FY)	Fenghuangluan Crab<br>(18--3)	Fuhong<br>(4--7)	Fujin<br>(BK-FJ)	Fushuai<br>(1--12)	Fuli<br>(QD-28)
Fuyan<br>(QD-21)	Fuxian Sanye<br>(XC-FXXY)	Fuga<br>(29--17)	Fu Hong<br>(25--5)	Fujion<br>(HS-6)	Fuqiu<br>(25--3)	Fuji<br>(BK-Fuji)	Fuji<br>(TA-9)
Gala × Mato 8<br>(YT-29)	Gaidebao<br>(9--7)	Ganhong<br>(YT-16)	Gao #5<br>(BK-G-5)	Gaoqiu<br>(BK-GQ)	Geliekekukui<br>(20--1)	Cooper's Market<br>(8--15)	Guoling<br>(1--15)
Guoqing<br>(24--13)	Guoqinghong<br>(YT-5)	Guoshuai<br>(5--19)	Haihong<br>(QD-19)	Haitangguo<br>(BK-HTG)	Haitanghua<br>(BK-HTH)	Hanfu 3<br>(GY-4)	Hanfu 6<br>(GY-3)
Hanfu 3x<br>(XC-3)	Hebei Kangbing Golden<br>(13--6)	Hebing Pingding Crab<br>(B2-14)	Heoersitai<br>(21--10)	Helasang<br>(12--23)	Khrushchev<br>(10--6)	Hesetiaowen<br>(12--3)	Todoroki Tsugaru<br>(9--12)
Hirosaki Fuji<br>(23--1)	Hong 4G<br>(B-3)	Hong Anka<br>(YT-41)	Red Baron<br>(22--2)	Hongbaoshi<br>(27--15)	Hongcuibao<br>(Z-9)	Gale Gala<br>(Z-10)	Pigeon<br>(18--13)
Hongguang<br>(WH-4)	Hong Crab<br>(B3-6)	Hong Crab 2<br>(BK-HHT2H)	Stark Redgold<br>(BK-HJ)	Hongjin Gala<br>(22--4)	Black Ben Davis<br>(10--1)	Calville Rouge<br>(12--14)	Red Astrachan<br>(3--9)
Hongli<br>(YT-49)	Honglingdang<br>(SY-21)	Doyle<br>(12--15)	Honglu seedling 65<br>(YT-93)	Hong Manao<br>(XYZ-1)	Hongmantang<br>(SX-24)	Red Jonagold<br>(BK-HQNJ)	Hongqiaowang<br>(24--17)

Red Jonaprince (HS-12)	Hongrou 1 (XN-HR1)	Hongrou 2 (XN-HR2)	Hongrou 3 (XN-HR3)	Hongrou 4 (XN-HR4)	Hongrou 5 (XN-HR5)	Hongrou 6 (XN-HR6)	Hongrou 7 (XN-HR7)
Hongrou Pingguo (15--9)	Hongsanye (XC-HSY)	Hongte (BK-HT)	Hongxia (27--8)	Hongxiangcui (BJ-9)	Cogswell Pearmain (17--17)	Starking (YT-25)	Hongxue (BK-HX)
Hongxun 1 (QD-22)	Jonathan (BK-Jonathan)	Kogetsu (15--17)	Hong Zhenzhu (Z-4)	Summer Champion (18--14)	Husveti Rosmaring (14--5)	Huadao (3--7)	Huaguan Crab (BK-HGHT)
Huahong (SY-19)	Wealthy (7--19)	Lowland Raspderry (3--3)	Huadan (31--2)	Huajia (Z-15)	Huamei (B3-12)	Huanong 1 (18--2)	Huarui (Z-8)
Huashuai (YT-36)	Huashuai 1 (5--21)	Huashuo (B3-13)	Huaxing (Z-31)	Huayu (31--8)	Huayue (B3-15)	Huaida (B-8)	Huangcui (WH-5)
Huangfu 7 (XY-44)	Yellow Fuji (28--18)	Huangguniang (2--5)	Harica (XY-90)	Huangtaiping (BK-HTP)	Huangtianguo (3--5)	Huifeng Orin (BK-HFWL)	Himekami (BK-JS)
Jierjisi (20--21)	Jilin Xiaohong Crab (XC-JILINXIAOH ONGHAITANG)	Very Early Fuji (29--14)	Jiping 1 (B-2)	Jiangxue (HS-8)	Jerseymac (1--7)	Jie 18 (7--16)	Jie 9 (18--4)
Jieba (21--2)	Jieernianke (28--9)	Golden Spur (26--14)	Golden Spur (YT-24)	Jincui (BK-JC)	Jinduhong Gala (YT-91)	Golden Delicious (BK-GD)	Jinguang (23--16)
Jinhong (BK-JH)	Jinhong (SY-6)	Willams Faborite (3--11)	Jinping (SY-10)	Jinse Luosuoshan (16--22)	Jinshiji (XYZ-7)	Jinshiji (Z-7)	Jinshuai mutant (YT-95)
Tsugaru (28--5)	Tsugaru (YT-94)	Jinnhong (12--5)	Jinxiu Crab (GZ-5)	Jinxiuhong (B-6)	Jin 18 (GY-1)	Jinfu 1 (XY-81)	Jingbohu Shandingzi (B3-2)

September Wonder Fuji<br>(HS-9)	Cox's Orange Pippin<br>(14--2)	Juda Fuji<br>(XY-37)	Cardinal<br>(9--20)	Karas Tor<br>(19--19)	Cameo<br>(31--3)	Kay Sai William<br>(12--6)	Cortland<br>(8--10)
Clapp's Seedling<br>(20--6)	Esopus Spitzenburg<br>(2--14)	KLGDG Shandingzi<br>(BK-KLGDGSDZ)	Kelia<br>(9--10)	Kelisike<br>(7--13)	Kelongxieer<br>(9--3)	Close<br>(1--19)	Cloden<br>(9--5)
Frequin Rouge<br>(23--15)	Kermerrien<br>(22--13)	Kuliesa<br>(10--21)	Kuluona<br>(13--13)	Kuihua<br>(3--22)	Sunflower<br>(BK-KUIHUA)	Apple of Commerce<br>(20--11)	Lobo<br>(11--13)
Lawfam<br>(18--20)	Laxtons Superb<br>(17--15)	Lanfengwang<br>(21--11)	Lenghaitang<br>(BK-LHT)	Lixing Crab<br>(YN-13)	Lysgolden<br>(10--10)	Lijiang Shandingzi<br>(BK-LJSDZ)	Lianji<br>(23--22)
Ryoka no Kisetsu<br>(BK-LX)	Liaofu<br>(2--7)	Liaozhen 1<br>(BK-LZ1H)	Liehuangjiatena<br>(3--15)	Linqin Crab<br>(LQ)	Linzhi<br>(XC-LZ)	Linyi Meiguo 1<br>(SX-34)	Linyi Meiguo 2<br>(SX-30)
Linyi Meiguo 4<br>(SX-4)	Linyi Meiguo 5<br>(SX-12)	Linyi Meiguo 6<br>(SX-3)	Linyi Meiguo 8<br>(SX-2)	Linyi Fuji<br>(XY-20)	Dolgo<br>(BK-DDG)	Lingyige Hongrou<br>(SX-18)	Liuyu mutant<br>(29--7)
Red June Sweet<br>(2--21)	Longfeng<br>(29--13)	Longfeng<br>(SY-12)	Longguan<br>(27--4)	Longguan<br>(SY-15)	Longhong<br>(29--15)	Longhong<br>(SY-23)	Longqiu<br>(SY-11)
Longshuai<br>(SY-16)	Longdong Crab<br>(XC-LDHT)	Lushi Crab<br>(XC-LSHT)	Lushan Sanye<br>(LSSY)	Ruby<br>(3--16)	Luli<br>(YT-98)	Luping 1<br>(TA-13)	Luping 2<br>(TA-14)
Luping 5<br>(TA-15)	Luyan<br>(TA-16)	Mutsu<br>(19--7)	Rutosh<br>(18--17)	Luxiang<br>(8--5)	Luanzhuang Crab<br>(B1-11)	Luanzhuang Shaguo<br>(B2-11)	Lord Lambourne<br>(17--9)
Lvguang<br>(21--6)	Lvshuai<br>(27--7)	Lvshuai<br>(B-4)	Lvxiangjiao<br>(BK-LXJ)	Saint Lawrence<br>(19--9)	Magu<br>(8--21)	Macoun<br>(2--9)	Malong Gala 2 blush<br>(YN-6)

Maiyan<br>(Z-25)	King of Pippin<br>(14--8)	Mao Shandingzi<br>(XC-MSDZ)	Meiduan 1<br>(7--10)	Melrose<br>(12--16)	Melba<br>(10--20)	Meltosh<br>(18--22)	America 8<br>(24--23)
Meile<br>(BK-ML)	Meilingxi Tsugaru<br>(8--13)	Marie Menard<br>(23--18)	Meinong<br>(TA-17)	Mensi<br>(15--5)	Kizashi<br>(21--20)	Mengpaisi<br>(BK-MPS)	Mitchgala<br>(SX-10)
Miqiulin Jinian<br>(3--4)	Mitchgla<br>(HS-3)	HoneyCrisp<br>(25--14)	Miguo<br>(28--3)	Honeygold<br>(5--3)	Mianpingguo<br>(12--9)	Mingyue<br>(YT-62)	Mollie's Delicious<br>(B-31)
Modi<br>(XYZ-10)	Morlie's<br>Delicious<br>(YN-18)	Moscow<br>Transparent<br>(8--14)	Mudanjiang<br>Crab<br>(B1-9)	Nanpu 2<br>(29--6)	Nanpu 3<br>(18--15)	Nic29<br>(HS-13)	Nimaiyisuo<br>(4--10)
Ningfeng<br>(26--23)	Ningguan<br>(24--15)	Ningqiu<br>(BK-NQ)	Milk<br>(SY-3)	Norand<br>(15--6)	Norda<br>(9--2)	Nvyoujidui<br>(2--16)	Nvyoujidui 2<br>(2--19)
Patten<br>(4--5)	Piga 101<br>(YT-63)	Piga 70<br>(YT-31)	Pinova<br>(29--16)	Pingyitiancha<br>(BK-PYTC)	Pingyitiancha<br>(XC-PYTC)	Pingyan<br>(CL-6)	Pulanhong<br>(YT-46)
Shichinohe 2<br>(XY-29)	Shichinohe 1<br>(25--9)	K9<br>(SY-7)	Qihe Golden Sper<br>(4--22)	Senshu<br>(BK-QQ)	Qianxue<br>(BK-QAINXUE)	Chantecler<br>(11--9)	Qianxian Crab<br>(QX-1)
Chimeric Ralls<br>Janet<br>(19--4)	Jonared<br>(15--18)	Jonagold<br>(5--1)	Joyal<br>(5--4)	Chenango<br>Strawberry<br>(17--4)	Qincui<br>(SX-15)	Qin'guan<br>(17--22)	Qinyang<br>(SX-6)
Qing n3<br>(29--2)	Qingdao 1<br>(6--13)	Qingfu 3<br>(QD-3)	Qingguan<br>(18--9)	Qinglin<br>(WH-6)	Qingping<br>(29--1)	Aomori Spur Fuji<br>(XY-46)	Aomori Early<br>(BK-QSZS)
Qingxiang<br>(25--6)	White Pearmain<br>(YT-70)	Seimei<br>(BK-QM)	Qiufengmi<br>(SY-20)	Akifu 39<br>(23--9)	Akifu 39<br>(TA-19)	Akifu 39<br>(XY-85)	Qiuhong<br>(YT-89)

Qiuhong Gala (BJ-8)	Qiuhong Gala (Z-40)	Qiulimu (SX-14)	Qiulimeng (18--16)	Qiulu (31--4)	Qiulu (SY-22)	Qiutianhong (9--6)	Qiuzi (XC-QZ)
Qiuxing Crab (BK-QXHT)	Qunfu 1 (14--23)	York Imperial (4--9)	Rushan Fuji (QD-23)	Judaine (25--11)	Judaine (TS-9)	Rome Beauty (17--11)	Jurella (23--20)
Juliana (26--5)	Retina (YT-85)	Ruixiang (7--18)	Judestar (26--9)	Judestar (TS-8)	Ruixue (ruixue)	Ruiyang (RY)	Sieversii (BK-SWS)
Saijin (QD-5)	Saiwen (4--17)	Sankuaishi Crab (B1-12)	Sankuaishi Crab 1 (BK-SKSHT1H)	Sankuaishi Crab 2 (B1-14)	Forest apple (BK-SLPG)	Summerland (4--20)	Shajin Crab (XC-JSHT)
Shanjin Crab N1 (QD-11)	Shanjin Crab N2 (QD-12)	Shajinyilamu (YT-88)	Shajin Yilamu (11--10)	Shalatuoni (2--4)	Shandingzi (XC-SDZ)	Shandingzi 2 (BK-SDZ2H)	Shandong 6 (Z-42)
Shanfu 2 (XY-89)	Sansa (BK-SX)	Chanteline (22--17)	Shenai LS (SX-19)	Shennong 2 (8--19)	Shengli Hongguan (10--14)	Shengfang 3A (XY-65)	Shizishan 1 (10--15)
Shidonghaoji (13--16)	Stonetosh (19--22)	Sekaiichii (26--18)	Shouer hong (YN-11)	Red Chief (TS-14)	Shou Fuji (XY-87)	Fengfeng Baleng (GY-2)	Shoufenshu 6 (XYZ-5)
Shuahong (19--12)	Shuangyanghong (QD-9)	Shuohong (31--17)	Spartan (11--2)	Stark Spur Golden (5--5)	Stark Gold (5--8)	Situonuowei (9--22)	Sichuan Bianye (XC-SCBY)
Soviet (11--11)	Sujsleppskoe (2--1)	Avrolles (23--17)	Taigu Shaguo late (SX-17)	Taiguo Shaguo Early (SX-7)	Taihong Gala (Z-38)	Pacific Rose (21--8)	Taishan Gala (Z-47)
Taishan Crab (YT-96)	Tangmutian (QD-10)	Tehong 2 (BK-YH2)	Tian Andongnuo (2--17)	Tian Andongnuo 2 (2--2)	Tianfeng (MDJ-9)	Douce Coetligne (26--10)	Tianhuangkui (3--13)
Douce Moen (26--2)	Tian Yisaye (8--17)	Turkmen apple (BK-TKMPG)	Wanbai Crab (BK-WBHT)	Wan Crab (B-1)	Orei (26--15)	Wangqiuhong (YT-45)	Wangshanhong (XY-80)

Wijcik McIntosh<br>(24--19)	Weiai 3<br>(BK-WA3)	Harlikar<br>(WH-8)	Weiqinni<br>(21--15)	Vista Bella<br>(4--16)	Weixi Sanye<br>(XC-WXSY)	Miki<br>(27--14)	Weixishengming<br>(25--8)
Weizhimuben<br>(XC-5)	Wengao 1<br>(YT-38)	Wengao 2<br>(YT-39)	Wengao 3<br>(YT-40)	Wengao 3 mutant<br>(YT-87)	Wenhong<br>(24--18)	Wuming 1<br>(BJ-1)	Smoothee<br>(21--17)
Wufengshan 1<br>(BK-WFS1H)	Wufengshan 4<br>(BK-WFS4H)	Wufengshan Crab<br>(BK-WFSHT)	Wufengshan Crab 2<br>(BK-WFSHT2H)	Wufengshan Crab 6<br>(BK-WFSHT6H)	Wuyue<br>(12--1)	Maigold<br>(8--20)	Wushan Bianye<br>(WSBY)
Polka<br>(29--11)	Maypole<br>(BK-WM)	Waltz<br>(BK-WZ)	Siberian White Spot<br>(3--2)	Xifu Crab<br>(XC-XFHT)	Watermelon Crab<br>(B-18)	Cellini<br>(8--23)	Simonffy Piros<br>(8--3)
Xite Shisheng<br>(8--16)	Xijin Crab<br>(BK-XJHT)	Xichang yuanzhuiguo<br>(XYZ-2)	Xiahong<br>(BH-1)	Xianhong<br>(YT-28)	Xiangguoguang<br>(14--11)	Xiangguo<br>(4--12)	Xiangjiaoguo<br>(SY-14)
Winter Banana<br>(20--14)	Xiangfu<br>(BJ-11)	Xiangyanghong<br>(BK-XYH)	Early Straw Berry<br>(8--9)	Xiaofan Crab<br>(B2-13)	Xiaofanshan Baleng<br>(B3-11)	Xiaofanshan Baleng 1<br>(B3-9)	Xiaofanshan Binzi<br>(BK-XFSBZ)
Xiaofanshan Crab<br>(BK-ZFSHT)	Xiaofanshan Crab 4<br>(BK-XFSHT4H)	Xiaogoumen Naizi<br>(BK-XGMNZ)	XGM Suan Binzi<br>(BK-XGMSBZ)	XGM Tian Binzi<br>(BK-XGMTBZ)	Xiaojin Bianye<br>(XC-XJBY)	Xiaojin Crab<br>(QD-8)	Xiaomian Crab<br>(BK-XMHT)
Xiaoshuai<br>(BK-XS)	Xin 1<br>(XC-1)	Xin 5<br>(XC-2)	Xindong<br>(16--14)	Xinhong<br>(16--1)	King David<br>(20--5)	Xinhua<br>(29--5)	Xinjiang 1<br>(BK-XJ1)
Xinjiang 11<br>(BK-XJ11)	Xinjiang 11<br>(XN-XJ11)	Xinjiang 13<br>(XN-XJ13)	Xinjiang 14<br>(XN-XJ14)	Xinjiang 15<br>(BK-XJ15)	Xinjiang 16<br>(BK-XJ16)	新疆 17<br>(XN-XJ17)	Xinjiang 18<br>(XN-XJ18)
Xinjiang 19<br>(XN-XJ19)	Xinjiang 2<br>(XN-XJ2)	Xinjiang 20<br>(XN-XJ20)	Xinjiang 21<br>(BK-XJ21)	Xinjiang 21<br>(XN-XJ21)	Xinjiang 22<br>(BK-XJ22)	Xinjiang 23<br>(XN-XJ23)	Xinjiang 24<br>(BK-XJ24)

Xinjiang 24<br>(XN-XJ24)	Xinjiang 25<br>(XN-XJ25)	Xinjiang 29<br>(BK-XJ29)	Xinjiang 3<br>(BK-XJ3H)	Xinjiang 3<br>(XN-XJ3)	Xinjiang 31<br>(BK-XJ31)	Xinjiang 4<br>(XN-XJ4)	Xinjiang 5<br>(XN-XJ5)
Xinjiang 6<br>(BK-XJ6H)	Xinjiang 7<br>(XN-XJ7)	Xinjiang 8<br>(BK-XJ8)	Xinjiang 8<br>(XN-XJ8)	Xinjiang 9<br>(BK-XJ9)	Xinjiang 9<br>(XN-XJ9)	Xinjiang<br>Hongrou Crab<br>(YT-12)	Xinjiang<br>Pingguo<br>(12--7)
Xinjiang<br>Yepingguo<br>(XC-XJYHT)	Xinliangxiang<br>(SX-27)	Shinsekai<br>(BK-XSJ)	New Redchief<br>(TS-2)	Xinyuanshuai<br>(29--3)	Sinano Red<br>(27--10)	Sinano Gold<br>(30--2)	Sinano Sweet<br>(28--2)
Xingping<br>(21--4)	Xiongyue Crab 1<br>(B1-13)	Xiongyue Crab 2<br>(B1-8)	Su E Shandingzi<br>(BK-SESDZ)	Xiushui<br>Guoguang<br>(19--3)	McIntosh<br>(11--18)	Yajiang Bianye<br>(XC-YJBY)	Yanhongmi<br>(BK-YHM)
Yan 6 Fenhong 143<br>(YT-84)	Yanfu 10<br>(YT-100)	Yanfu 2<br>(YT-42)	Yanfu 3<br>(YT-14)	Yanfu 4<br>(YT-69)	Yanga1<br>(Z-12)	Yanzhen 1<br>(YT-32)	Iwaki<br>(17--21)
Yanyuan 1<br>(SC-1)	Yanyuan 4<br>(SC-4)	Yanyuan 5<br>(SC-5)	Yanyuan 6<br>(SC-6)	Yanyuan 7<br>(SC-7)	Yanshanhong<br>(23--13)	Yoko<br>(BK-YG)	Yangbai Crab<br>(YT-6)
Evelyn<br>(9--21)	Iran Pippin<br>(10--4)	Yishuihong<br>(TA-32)	Canzy ?<br>(BJ-10)	Alps Otome<br>(TA-27)	Italy Smothe<br>(WH-10)	Italy Early Red<br>(BK-YDLZH)	Silver Spur Red<br>Delicious<br>(11--4)
Indo<br>(28--0)	Indo<br>(YT-26)	Ingram<br>(20--7)	Akin's Red<br>(18--21)	Cherry Crab<br>(XY-6)	Yingye Crab<br>(XC-YYHT)	Yingqiu<br>(9--23)	Prime Gold<br>(10--9)
Youlixiang<br>(25--21)	Youyi<br>(7--23)	Grimes Golden<br>(8--8)	Yuanhong<br>(BK-YH)	Yueguan<br>(XY-10)	Yuehua<br>(XY-11)	Yueli<br>(XY-88)	Yuemei<br>(XY-18)
Yueping<br>(XY-60)	Yueshuai<br>(XY-28)	Yueyan<br>(XY-12)	Yueyanghong<br>(31--15)	Yun Hongrou<br>(YN-12)	Zaobai Crab<br>(B3-1)	Zaohong<br>(4--11)	Early Red Bird 2<br>(BJ-7)

Zaohongda Gala (27--6)	Zaohongxia (27--9)	Early Harvest (1--13)	Geneva Early (BK-ZJ)	Early Golden (2--8)	Wase16 (15--16)	Early Red Bird (4--6)	Early Jonagold (YT-65)
Early McIntosh (9--18)	Early Worcester (3--23)	Jersey Mac (Z-1)	Zhaai (XC-ZA)	Zach Lebel (8--1)	James Grieve (1--23)	Zhanhanxiang (3--19)	Zhanxuan 6 (16--10)
Zhangye 2 (31--12)	Nagafu 3-R (BK-3)	Nagafu 6 (BK-CF6H)	Changguo Crab (B-14)	Changhong (17--16)	Nagafu 1 (10--12)	Stark Jumbo (BK-ZB)	Jumbo Orin (BK-ZBWL)
Zhengding 2 (3--21)	Zhongqiu (8--6)	Zhongqiuwang Linyi (SX-11)	Zhongxing (25--7)	Zhongnvshi (QD-26)	Chongban Crab (XY-9)	Zumi Crab (XC-ZMHT)	Zumi Crab 3x (B-15)
Zumi Crab 3x 2 (B-22)	Zumi Crab W1 (B-29)	Summer Pearmain (11--20)	Zhuifeng 1 (BK-ZF1H)	Zhuifeng 2 (BK-ZF2H)	Zisai Pearl (BK-Zisai)	Drumbo (20--17)	Zixiang (SY-17)
Black Gilliflower (4--1)	Ziye zidaguo (XYZ-4)	Ziye zixiaoguo (XYZ-3)	Lowver (1--3)	Whitney (3--1)	Helm (11--21)	Miyazaki Spur Fuji (BK-GQDZ)	2001 Spur (QD-27)
Shengfang (TA-22)	KAKUFUJI (XY-30)	Shengfang 2 (BK-SF2)	Fubrax (HS-2)	Yishui Fuji (XY-22)	Mouping guanghua Fuji (YT-8)	Fukushima Spur Fuji (BK-FDDZ)	Fujiko (HS-4)
Yanfu 6 (QD-32)	Starkrimson (BK-XHX)	HardiSpur Delicious (6--21)	Ruby (BK-Ruby)	Royal Gala (Z-50)	Gala Queen (Z-27)	Cherry Gala(YN-9)	Malong Gala 2(YN-5)
Malong Gala 1(YN-1)	Yanga (Z-34)	Sweet Jonathan (20--10)	Jonathan-early (YT-73)	Spur Golden Delicious (BK-DJG)	Fangming (BK-FM)	Red Ralls Janet (BK-HGG)	Ralls Janet (19--2)
Sweetie (YN-7)	Starkjam (9--9)	Freybreg (14--4)	Ben David (19--8)	row 3 (YN-25)	Jiguan (BK-JG)	Yuanye Crab (BK-YYHT)	Sweet McIntosh (11--16)

Red Canada<br>(13--2)	Calville Blanche<br>(14--3)	Gravenstein<br>(1--6)	Zhuoai 1<br>(16--2)	Northern Spy<br>(17--10)	Newfane<br>(17--7)	Campbell<br>(18--11)	Xinlimei<br>(18--19)
Giant Jeniton<br>(19--1)	Radiant<br>(20--23)	Boskoopske<br>Cervene<br>(2--11)	Hongzhiwu<br>(21--18)	Judeline<br>(25--12)	Ningguang<br>(25--19)	Fameuse<br>(3--18)	Daguo Crab<br>(B-17)
Ambrosia<br>(B-27)	Luli<br>(B-5)	Kokyu<br>(26--19)	Laoshan 4<br>(BK-LS4H)	Yanqing<br>(BK-QY)	Mato 1<br>(BK-TMYH)	Stark Blushing<br>Golden<br>(BK-JY)	Kotoku<br>(BK-XD)
Xinjiang 14<br>(BK-XJ14)	Xinjiang 17<br>(BK-XJ17)	Xinjiang 19<br>(BK-XJ19)	Hanfu<br>(SY-4)	Chaguo<br>(XC-CG)	Jilin Huang Crab<br>(XC-JLHHT)	Xinjiang 10<br>(XN-XJ10)	Xinjiang 15<br>(XN-XJ15)
Xinjiang 16<br>(XN-XJ16)	row 20<br>(YN-40)	Freedom<br>(YT-80)	51-139<br>(YX-51-139)	Zaocuilv<br>(22--16)	CG80<br>(BK-CG80)	Chieftan<br>(BK-chieftan)	Zumi Crab<br>(B-30)
Ruihong<br>(QD-25)	New Jonagold<br>(BK-XQNJ)						
