# Supplementary figures and images for: Application of genome-wide insertion/deletion markers on genetic structure analysis and identity signature of Malus accessions
Source: BMC Plant Biol. 2020 Nov 30;20:540. doi: 10.1186/s12870-020-02744-2 (PMC7708918; doi:10.1186/s12870-020-02744-2)

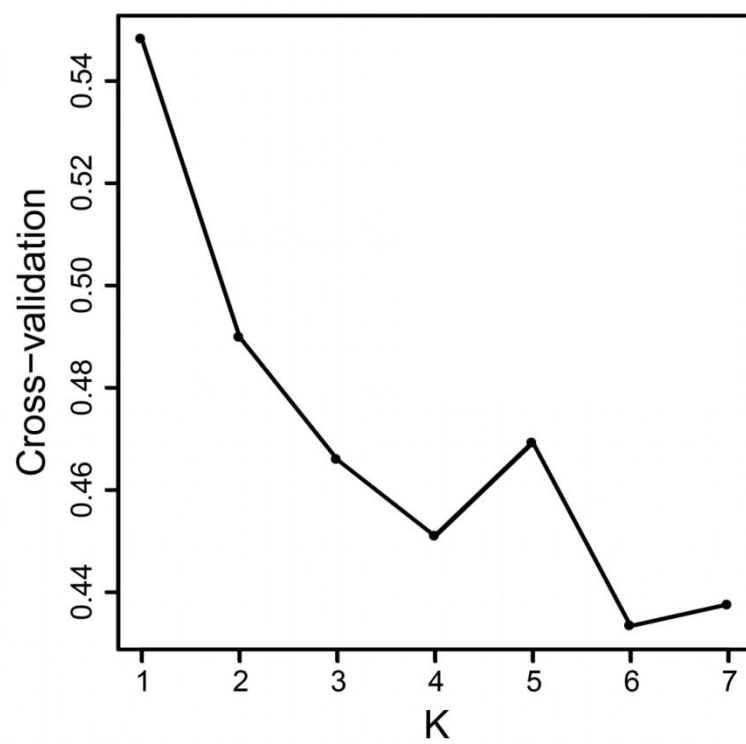

**Figure S1** Cross-validation plot for the InDel dataset.

Supplement: Supplementary file 11 — Additional file 11: Supplementary Figure S1. Cross-validation plot for the InDel dataset. [file 12870_2020_2744_MOESM11_ESM.pdf]
